# Nerve agent exposure and physiological stress alter brain microstructure and immune profiles after inflammatory challenge in a long-term rat model of Gulf War Illness

**DOI:** 10.1016/j.bbih.2024.100878

**Published:** 2024-09-30

**Authors:** Chia-Hsin Cheng, Yi Guan, Vidhi P. Chiplunkar, Farzad Mortazavi, Maria L. Medalla, Kimberly Sullivan, James P. O'Callaghan, Bang-Bon Koo, Kimberly A. Kelly, Lindsay T. Michalovicz

**Affiliations:** a Chobanian & Avedisian School of Medicine, Boston University, Boston, MA, USA; b School of Public Health, Boston University, Boston, MA, USA; c Guest Researcher, Health Effects Laboratory Division, Centers for Disease Control and Prevention – National Institute for Occupational Safety and Health, Morgantown, WV, USA; d Health Effects Laboratory Division, Centers for Disease Control and Prevention – National Institute for Occupational Safety and Health, Morgantown, WV, USA

**Keywords:** Gulf War Illness, Neuroinflammation, Diffusion MRI, Glial activation, DFP, CORT

## Abstract

Gulf War Illness (GWI) is a disorder experienced by many veterans of the 1991 Gulf War, with symptoms including fatigue, chronic pain, respiratory and memory problems. Exposure to toxic chemicals during the war, such as oil well fire smoke, pesticides, physiological stress, and nerve agents, is thought to have triggered abnormal neuroinflammatory responses that contribute to GWI. Previous studies have examined the acute effects of combined physiological stress and chemical exposures using GWI rodent models and presented findings related to neuroinflammation and changes in diffusion magnetic resonance imaging (MRI) measures, suggesting a neuroimmune basis for GWI. In the current study, using *ex vivo* MRI, cytokine mRNA expression, and immunohistological analyses of brain tissues, we examined the brain structure and immune function of a chronic rat model of GWI. Our data showed that a combination of long-term corticosterone treatment (to mimic high physiological stress) and diisopropyl fluorophosphate exposure (to mimic sarin exposure) primed the response to subsequent systemic immune challenge with lipopolysaccharide resulting in elevations of multiple cytokine mRNAs, an increased activated glial population, and disrupted brain microstructure in the cingulate cortex and hippocampus compared to control groups. Our findings support the critical role of neuroinflammation, dysregulated glial activation, and their relationship to disrupted brain microstructural integrity in the pathophysiology of 10.13039/501100008602GWI and highlight the unique consequences of long-term combined exposures on brain biochemistry and structural connectivity.

## Highlights

• LPS exacerbated brain and liver inflammation after chronic CORT and DFP exposure.
• Diffusion MRI captured consistent group patterns with inflammatory cytokines.
• GFA patterns further correlated with glial activation markers from brain tissues.

## 1 Introduction

Gulf War Illness (GWI) refers to the multi-symptomatic disorder experienced by one-third of 1991 Gulf War veterans (White et al., 2016). Evidence suggests exposures to toxicants during the war, such as oil well fire smoke, pesticides, nerve agents, as well as environmental conditions like heat and physiological stress, may trigger abnormal neuroinflammatory responses that contribute to GWI pathophysiology (White et al., 2016; Sullivan et al., 2018; Garza-Lombó et al., 2021). Our previous work examined the effect of combined high physiological stress and GW-relevant nerve agent using GWI rodent models (O'Callaghan et al., 2015; Ashbrook et al., 2018; Locker et al., 2017; Koo et al., 2018; Michalovicz et al., 2019). We have shown that prior subchronic stress hormone (corticosterone, CORT) exposure exacerbated the neuroinflammatory responses to subsequent acute diisopropyl fluorophosphate (DFP, nerve agent surrogate) challenge, as well as producing subtle but significant changes in brain structural integrity in the absence of significant neuronal damage (O'Callaghan et al., 2015; Koo et al., 2018). Specifically, diffusion magnetic resonance imaging (dMRI) revealed significantly increased microscale diffusivity in the frontal cortex and hippocampus following CORT and CORT + DFP exposures compared to controls, indicating potential neuroinflammatory-driven cellular morphological changes (Koo et al., 2018). Moreover, we found that the initial CORT + DFP exposure, reflective of “in-theatre” exposure conditions, exacerbated the brain response to future systemic inflammatory challenge in mice (Michalovicz et al., 2021). These results suggest that the initiating exposures experienced during deployment have the potential to perpetuate long-term changes in the neuroimmune response, highlighting the unique consequences of combined GW-relevant chemical and environmental exposures on the brain.

Brain imaging techniques are useful tools for evaluating the effects of various exposures and monitoring neuroinflammation *in vivo*. Positron emission tomography (PET) using translocator protein, TSPO, has shown direct evidence of enhanced neuroinflammation and glial activation in veterans with GWI, which was further captured by dMRI (Alshelh et al., 2020; Cheng et al., 2021). While PET imaging is limited by technical feasibility, MRI is a more accessible, less invasive tool to examine brain structural changes underlying GWI. Structural MRI analysis has shown reductions in global and regional gray and white matter volumes in veterans with GWI compared to healthy veterans, which may be associated with alterations in brain anatomical connectivity measured by dMRI (Chao et al., 2011). These previous findings indicate a potential involvement of astrocytes and microglia in GWI neuropathology; recent advances in dMRI may enable more precise investigation of astrocyte and microglia activation *in vivo* (Garcia-Hernandez et al., 2022).

In order to evaluate the long-term consequences of combined exposures to high physiological stress and nerve agent on immune responses and brain structure, we recapitulated our long-term GWI mouse model in rats to expand upon our previous *ex vivo* imaging studies. Here, we evaluated brain and liver cytokine mRNA expression to assess neuroinflammation and reconstructed microscale restricted diffusivity (micro-D) and generalized fractional anisotropy (GFA) to provide quantitative measures in the previously identified localized regions of interest (ROI), the anterior cingulate cortex and hippocampus (Yeh et al., 2010; Koo et al., 2018).

## 2 Materials and methods

**Fig. 1:**
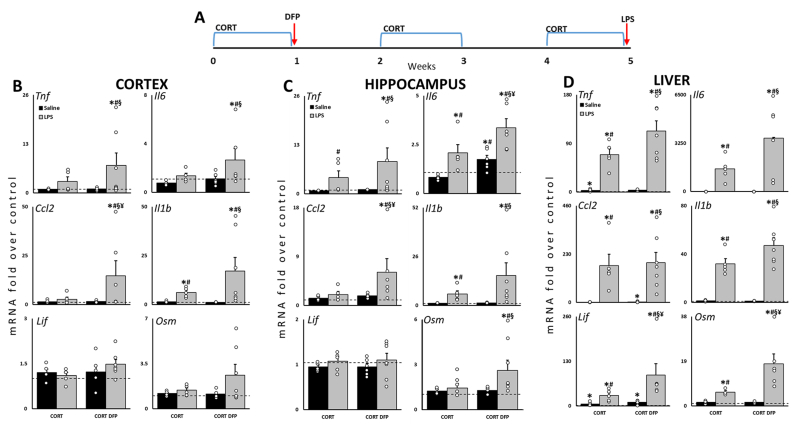
**Prior exposure to CORT and DFP increases the expression of cytokine mRNAs in response to subsequent challenge with LPS.** (**A**) Rats were exposed to CORT for 7d followed by DFP on Day 8. Rats were re-exposed to bouts of CORT every other week for 4 weeks and challenged with LPS on Day 36. *Tnf*, *Il6*, *Ccl2*, *Il1b*, *Lif*, and *Osm* mRNA levels were measured in cortex (**B**), hippocampus (**C**) and liver (**D**) at 6h post-LPS by qPCR. Data represents mean ± SEM (N = 5–7/group); dashed line indicates saline control value and open circles indicate individual data points. *P* ≤ 0.05 compared to saline control(∗), CORT(#), CORT + DFP(§), or CORT + LPS(¥).

Detailed methods for animal dosing, tissue collection, and assays are described in Supplementary Materials. Animal procedures were performed within protocols approved by the Centers for Disease Control and Prevention-Morgantown Institutional Animal Care and Use Committee and the US Army Medical Research and Development Command Animal Care and Use Review Office in an AAALAC International accredited facility. Briefly, rats were exposed to CORT in the drinking water for 7 days followed by a single injection of DFP then re-exposed to CORT every other week followed by an LPS challenge (Fig. 1A). Brain and liver tissues were collected and frozen at 6h post-exposure for mRNA analysis and whole brains were collected after perfusion at 24h post-exposures for MRI and immunohistochemistry.

To assess cytokine expression, quantitative PCR (qPCR) analysis of total RNA was performed on the frontal cortex, hippocampus, and liver as previously described (Locker et al., 2017). Specifically, we analyzed tumor necrosis factor alpha (*Tnf*), interleukin-6 (Il6), C-C chemokine ligand 2 (*Ccl2*), interleukin 1β (*Il1b*), leukemia inhibitory factor (*Lif*), and oncostatin M (*Osm*), along with the housekeeping gene, glyceraldehyde-3-phosphate dehydrogenase (*Gapdh*). Data is available on the NIOSH Data and Statistics Gateway (https://www.cdc.gov/niosh/data/).

Diffusion MRI was performed on a 4.7T MRI with an applied diffusion weighted spin-echo echo planar imaging sequence (SE-EPI) and restricted diffusivity (micro-D) and generalized fractional anisotropy (GFA) maps were reconstructed as previously described to detect changes related to cell density and glial cell activities (Koo et al., 2018).

Immunostaining was performed on PFA-fixed, frozen brain sections (70 μm coronal slices). Microglia and astrocytes were immunolabelled with ionized calcium binding adaptor molecule 1 (denoted Iba1+) and glial fibrillary acidic protein (denoted GFAP+), respectively, imaged, and classified into activated and homeostatic. Raw MRI and immunostaining data are available through the Boston Biorepository, Recruitment and Integrated Network for GWI (BBRAIN) via proposal submission to the steering committee (https://sites.bu.edu/bbrain/).

For qPCR, one-way ANOVAs were conducted on log-transformed values using SigmaPlot v15, followed by Fisher LSD post-hoc analysis (*P* ≤ 0.05). For dMRI and cell counting, group-level comparisons were performed in MATLAB (R2022a) using student t-test (*P* ≤ 0.05) followed by multiple comparison corrections using Benjamini Hochberg FDR, as used previously (Koo et al., 2018). The association between MRI and cell count was analyzed using Pearson correlation analysis.

## 3 Results

### 3.1 Changes in cytokine mRNA expression in the long-term rat model of GWI

Previously, we developed a cytokine mRNA panel comprising Th1 and gp130 cytokines to evaluate the neuroinflammatory effects of neurotoxicant exposure (O'Callaghan et al., 2008; O'Callaghan et al., 2014), which we have utilized to evaluate our GWI rodent models. Using this panel, we found several cytokine mRNAs significantly elevated following LPS treatment in the cortex and hippocampus (Fig. 1). Specifically, the CORT + DFP + LPS group showed higher *Tnf*, *Il6, Ccl2,* and *Il1b* in cortex (Fig. 1B) and higher *Tnf*, *Il6*, *Ccl2, Il1b*, and *Osm* in hippocampus (Fig. 1C), compared to Saline, CORT, and CORT + DFP groups. Cortical and hippocampal *Ccl2* and hippocampal *Il6* were significantly different compared to CORT + LPS. Unsurprisingly, LPS induced significant peripheral inflammation indicated by the significant elevation of cytokine mRNA levels in liver (Fig. 1D). Liver *Lif* and *Osm* expression were significantly increased by CORT + DFP + LPS exposure compared to CORT + LPS.

### 3.2 Changes in dMRI microstructural diffusivity in the cingulate and hippocampus

**Fig. 2:**
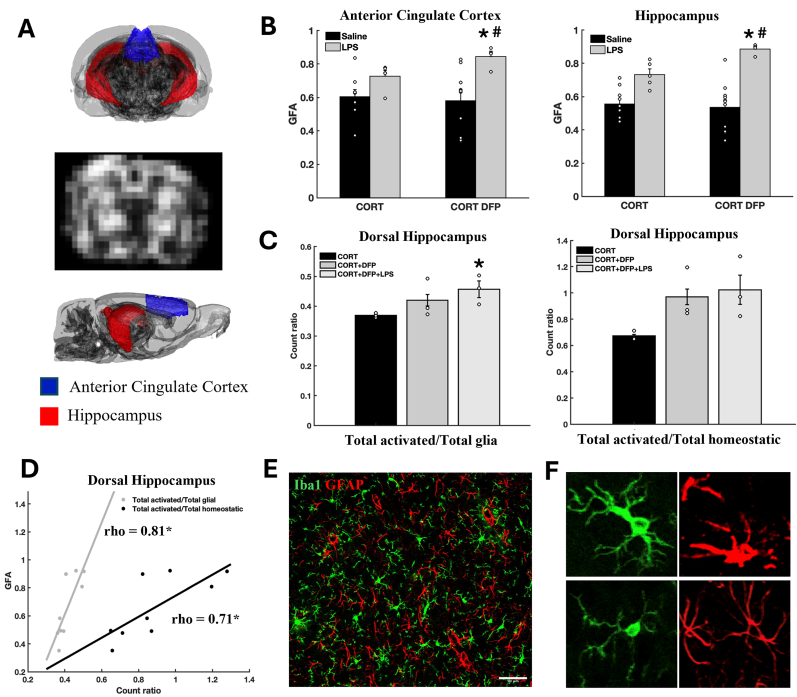
**Effects of CORT, DFP and LPS on brain microstructural integrity. A**: 3D rat brain showing ROIs and a representative GFA map. **B**: Group-level comparison of GFA in each ROI comparing three exposure groups to saline control. Open circles indicate individual data points. **C**: Quantitative analysis of total activated glia (astrocytes and microglia) to total glia ratio among three exposure groups. Open circles indicate individual data points. **D**: Correlation between GFA and glial cell count ratio in left dorsal hippocampus. **E**: Representative image of Iba1+ microglia (green) and GFAP + astrocytes (red) in the left dorsal hippocampus. **F**: Representative images of activated (top left) and homeostatic microglia (bottom left), reactive (top right) and homeostatic (bottom right) astrocytes. *P* ≤ 0.05 compared to the CORT(∗) or CORT + LPS(#) group. (For interpretation of the references to colour in this figure legend, the reader is referred to the Web version of this article.)

GFA mapping demonstrated similar group patterns as observed in inflammatory cytokine profiles. Specifically, CORT + DFP + LPS exposure showed higher GFA in both the cingulate cortex and hippocampus, compared to CORT and CORT + LPS (Fig. 2B). While we previously reported that micro-D mapping was able to differentiate exposure groups in the acute model (Koo et al., 2018), here, we did not observe significant differences across exposure groups for micro-D mapping (Supplementary Fig. 1). However, we observed an overall significantly higher micro-D in all exposed groups (CORT, CORT + DFP, and CORT + DFP + LPS) compared to saline control in both the cingulate cortex and hippocampus (Supplementary Fig. 1).

### 3.3 Changes in glial activation in dorsal hippocampus

When comparing total glial cell populations, astrocytes and microglia combined, in the left dorsal hippocampus, we found a significantly higher ratio of total activated glial cells to total glial cells in the CORT + DFP + LPS group compared to CORT (Fig. 2C). When examining the correlation between dMRI and cell counts, we found that higher GFA in hippocampus was significantly correlated with the ratio of activated glial cells to total glial cells or total homeostatic glial cells (Fig. 2D).

## 4 Discussion

In the current study, we examined cytokine and brain structural profiles in a chronic GWI rat model finding that: 1) prior exposure to CORT + DFP exacerbated the cytokine response to subsequent LPS challenge in the cortex, hippocampus, and liver, 2) CORT + DFP + LPS exposure disrupted GFA brain diffusivity measures in the cingulate cortex and hippocampus, 3) CORT + DFP + LPS exposure increased the proportion of activated microglia and astrocytes in CA1 of the hippocampus. These results corroborate previous studies suggesting a critical role of neuroinflammation, dysregulated glial activation, and their relationships to disrupted limbic and hippocampal microstructural integrity in GWI (Koo et al., 2018; Madhu et al., 2019; Alshelh et al., 2020; Cheng et al., 2020; Garza-Lombó et al., 2021).

The enhanced brain cytokine response observed in the chronic GWI rat model was consistent with previous findings from our acute GWI rodent models and the chronic GWI mouse model (O'Callaghan et al., 2015; Koo et al., 2018; Michalovicz et al., 2021) and may better recapitulate the long-term conditions experienced by veterans with GWI (Carrera Arias et al., 2021). While LPS treatment increased cytokine mRNA expression in the brain and liver, rats previously exposed to CORT + DFP showed a pattern of exacerbated cytokine mRNA levels compared to CORT + LPS. This observation contrasts with our previous findings in the acute GWI mouse model, where prior exposure to CORT exacerbated the DFP-induced expression of brain cytokines while normalizing or reducing the expression of liver and serum cytokines (O'Callaghan et al., 2015; Michalovicz et al., 2019). The CORT-induced exacerbation of brain cytokine expression is paradoxical considering its inherent anti-inflammatory effects (Coutinho and Chapman 2011) and the role of endogenous corticosterone in modulating inflammatory responses (Goujon et al., 1997). However, several studies have demonstrated the inflammatory priming effects of chronic exogenous CORT exposure or stress-elevated CORT (de Pablos et al., 2006; Sorrells and Sapolsky, 2007; Frank et al., 2014; Kelly et al., 2018). The temporospatial differences in cytokine expression observed between our acute and long-term GWI models are interesting and suggest potential differential effects of CORT on DFP and LPS. Specifically, while one week of CORT exerts more traditional anti-inflammatory effects on peripheral responses to DFP (Michalovicz et al., 2019), it largely exacerbates the liver cytokine mRNA response to LPS exposure in mice (unpublished data). With long-term CORT exposure, LPS-induced expression of brain and liver cytokines are both exacerbated compared to LPS alone (Kelly et al., 2018; unpublished data), similar to what is observed in the present study. This suggests that the mechanisms by which glucocorticoid signaling interacts with DFP- and LPS-induced inflammatory and/or other signaling pathways may diverge. Additionally, it is unclear if prior CORT + DFP exposure may directly activate other pathways in the periphery or exert effects on the periphery through aberrant neuroimmune signaling that prime these future responses to inflammatory challenge. Further investigation into these mechanisms may provide greater insight into how nerve agents produce neuroinflammation and disease, as well as highlight unique pathways that may be targeted for treatment intervention. It is important to note that a caveat to these interpretations is that our tissues collected for qPCR were not saline-perfused to avoid confounding effects of anesthetic agents on the brain and, thus, the results may represent a mixture of tissue-specific and blood cytokine responses. While further work is needed, our results correlate with studies in veterans with GWI that demonstrated elevated peripheral immune responses when challenged (Broderick et al., 2013; Van Booven et al., 2021).

Cytokines function as immune mediators and can have various activities within the immune system. Here, we have used a previously established set of cytokines to evaluate the neuroimmune response to neurotoxic exposures. These cytokines are largely associated with proinflammatory processes, including the activation of NF-κB and STAT3, the release of additional cytokines, and activation of microglia, yet several studies have indicated a potential role for these cytokines in anti-inflammatory signaling, particularly LIF and OSM (Janssens et al., 2015; Metcalfe 2019). Taken alone, our results are indicative of active neuroimmune signaling but not necessarily of neuroinflammation. Taken in concert with increases in activated microglia and astrocytes, our findings provide stronger support for a neuroinflammatory phenotype instigated by our combined 10.13039/501100008602GWI exposure. However, considering the dual inflammatory/protective nature of both cytokines and glia (Chen and Trapp, 2016; Escartin et al., 2021), further investigation is needed to clarify the role of specific cytokines, as well as the role of microglia and astrocytes, in GWI.

Our dMRI data demonstrated subtle changes in brain subcellular components following GWI-relevant exposures. The GFA measure captured diffusivity changes from the LPS-treated groups, which was further correlated with increased numbers of activated microglia and astrocytes and provided an indirect link between brain dMRI patterns and cytokine profiles, while the micro-D measure reflected overall changes in tissue microstructure and distinguished exposed groups from the saline control, as reported previously (Koo et al., 2018). This suggests that GFA and micro-D may have different sensitivity to inflammatory cellular changes in the gray matter of the brain and, thus, may require additional validation for better interpretation; GFA may better capture subtle morphological changes associated with microglia and astrocytes at different inflammatory stages (Garcia-Hernandez et al., 2022).

There are a few limitations of the current analysis that should be considered in future studies. First, this study focused on adult male rats, as most veterans with GWI are male. We hope to examine sex differences in the future as increasing evidence from GW veteran studies suggest an underlying sex effect (Heboyan et al., 2019; Sullivan et al., 2020). Second, the current study was largely driven by our hypothesis that the neuroimmune dysfunction associated with GWI is largely driven by astrocyte and microglia responses. However, there is also the potential for underlying neuron and/or oligodendrocyte dysfunction to impact GWI symptoms (Rao et al., 2017; Belgrad et al., 2019). Future research will focus on addressing the complex interactions between different cell types across multiple brain regions.

In conclusion, our chronic GWI model recapitulates the neurological component likely underlying persistent neuroimmune disruption and symptom flare-ups experienced by veterans with GWI. Previously, we have demonstrated the translational value of evaluating specific MRI techniques in animal models of GWI for subsequent use for human assessment (Cheng et al., 2020, 2021). Here, the combined evaluation of MRI, brain cytokine expression, and histology in our long-term GWI rodent model provides a framework by which the MRI results can be interpreted and validated to detect specific structural changes to certain cell types. As these brain cytokine and histological assays cannot be done in living veterans, the work presented here can be translated to provide interpretation of similar dMRI analyses in veterans with GWI.

## CRediT authorship contribution statement

**Chia-Hsin Cheng:** Writing – review & editing, Writing – original draft, Visualization, Validation, Methodology, Investigation, Formal analysis, Data curation. **Yi Guan:** Writing – review & editing, Writing – original draft, Visualization, Validation, Investigation, Formal analysis. **Vidhi P. Chiplunkar:** Validation, Investigation, Formal analysis, Data curation. **Farzad Mortazavi:** Writing – review & editing, Validation, Investigation. **Maria L. Medalla:** Writing – review & editing, Validation, Methodology. **Kimberly Sullivan:** Writing – review & editing, Supervision, Project administration, Conceptualization. **James P. O'Callaghan:** Writing – review & editing, Supervision, Resources, Project administration, Methodology, Funding acquisition, Conceptualization. **Bang-Bon Koo:** Writing – review & editing, Writing – original draft, Validation, Supervision, Software, Resources, Project administration, Methodology, Funding acquisition, Conceptualization. **Kimberly A. Kelly:** Writing – review & editing, Visualization, Validation, Supervision, Resources, Project administration, Methodology, Investigation, Funding acquisition, Formal analysis, Data curation, Conceptualization. **Lindsay T. Michalovicz:** Writing – review & editing, Visualization, Validation, Supervision, Resources, Project administration, Methodology, Investigation, Funding acquisition, Formal analysis, Data curation, Conceptualization.

## Declaration of competing interest

The authors declare the following financial interests/personal relationships which may be considered as potential competing interests:

Bang-Bon Koo reports financial support was provided by US Office of 10.13039/100000090Congressionally Directed Medical Research Programs. If there are other authors, they declare that they have no known competing financial interests or personal relationships that could have appeared to influence the work reported in this paper.

## Data Availability

Some data is available via the database linked in the manuscript, the other data is available upon request.
