# Supplementary material for: Nerve agent exposure and physiological stress alter brain microstructure and immune profiles after inflammatory challenge in a long-term rat model of Gulf War Illness
Source: Brain Behav Immun Health. 2024 Sep 30;42:100878. doi: 10.1016/j.bbih.2024.100878 (PMC11489046; doi:10.1016/j.bbih.2024.100878)
Supplement: Multimedia component 1 [file mmc1.docx]

**Nerve agent exposure and physiological stress alter brain microstructure and immune profiles after inflammatory challenge in a long-term rat model of Gulf War Illness**

*Chia-Hsin Cheng^a,1^, Yi Guan^a,1^, Vidhi P. Chiplunkar^a^, Farzad Mortazavi^a^, Maria L. Medalla^a^, Kimberly Sullivan^a,b^, James P. O’Callaghan^c^, Bang-Bon Koo^a^, Kimberly A. Kelly^d^, and Lindsay T. Michalovicz^d,^**

*^a^Chobanian & Avedisian School of Medicine, Boston University, Boston, MA, USA*

*^b^School of Public Health, Boston University, Boston, MA, USA*

*^c^Guest Researcher, Health Effects Laboratory Division, Centers for Disease Control and Prevention – National Institute for Occupational Safety and Health, Morgantown, WV, USA*

*^d^Health Effects Laboratory Division, Centers for Disease Control and Prevention – National Institute for Occupational Safety and Health, Morgantown, WV, USA*

***^1^equal contributions***

****corresponding author:*** [*yqp4@cdc.gov*](mailto:yqp4@cdc.gov) 1000 Frederick Ln, M/S L3014, Morgantown, WV 26508

**Supplemental Methods**

**Animal dosing:** Upon arrival, adult male Sprague Dawley rats (N=68; 250-300g; Hilltop Lab Animals, Scottdale, PA, USA; RGD_70508) were single housed in the CDC-Morgantown Animal Facility and allowed to acclimate for at least one week prior to the exposures. Rats were given food and water ad libitum and received daily health checks from animal husbandry personnel. Rats were exposed to CORT (200mg/L in 0.6% EtOH; Steraloids, Newport, RI, USA) in the drinking water for 7 days followed by a single injection of DFP (1.5mg/kg, i.p; MilliporeSigma, St. Louis, MO,USA) on Day 8. Following this initiating exposure, rats were re-exposed to CORT every other week for 4 weeks and then challenged with LPS (0.25 mg/kg, s.c.; L4516 from E. coli O127:B8l; MilliporeSigma) on Day 36. Before dosing, rats were randomly assigned to one of six groups and separated into two cohorts: sacrifice by decapitation at 6h for quantitative PCR (qPCR) or terminal-formalin perfusion at 24h for MRI or immunostaining (**Supplementary Table 1**).

**Tissue preparation:** For qPCR, brains were removed from the skull and frontal cortex and hippocampus were dissected, as previously described (Michalovicz et al., 2021); a small portion of one liver lobe was collected. Tissues were immediately frozen on dry-ice and stored at -80°C until qPCR. For MRI and immunostaining, rats were deeply anesthetized with Fatal Plus (300mg/kg, i.p.; Vortech Pharmaceuticals, Dearborn, MI, USA) and transcardially-perfused with 1x phosphate buffered saline (PBS) followed by 4% paraformaldehyde (PFA; FD Neurotechnologies, Columbia, MD, USA) using a peristaltic pump (8-10 mL/min flow rate; 70-7000C; Harvard Apparatus, Holliston, MA, USA). Whole brains were collected and stored in 4% PFA for further analysis.

**Cytokine profiling:** qPCR analysis of total RNA was performed using an ABI7500 Real-Time PCR System (ThermoFisher Scientific, Waltham, MA, USA) and TaqMan® chemistry, as previously described (Locker et al., 2017). mRNA expression level changes were calculated using the ΔΔCt method with normalization to *Gapdh* and expressed as fold-change over corresponding saline controls.

**MRI acquisition and processing:** SE-EPI was acquired using a 4.7T Bruker MRI (Billerica, MA, USA) with the following parameters: 586 μm isotropic voxel, coronal slice acquisition with 515 diffusion gradient directions using b-values up to 40,000 s/mm2 (Wedeen et al., 2008). Five non-diffusion weighted (b0) images were averaged to perform pre-processing of raw diffusion scans, and processed with modified in-house processing pipeline (Koo et al., 2018). All diffusion maps for each brain were nonlinearly transformed to the SIGMA rat brain atlas space to perform group-level statistics (Barrière et al., 2019) and permutation tests corrected for possible random effects (Koo et al., 2018).

**Immunostaining and confocal imaging:** Immunostaining was performed on PFA-fixed, cryoprotected, free-floating, 70μm coronal brain sections. After washing in 0.01M PBS, samples were incubated for 1h in blocking solution at room temperature (0.5% SuperBlock, 1M glycine, 0.4% TritonX) followed by 48h with primary antibodies at 4°C (Goat Anti-Iba1, 1:500, Abcam, Cambridge, UK, AB_2224402; Rabbit anti-GFAP, 1:500, Abcam, AB305808) with a short incubation in a low-wattage microwave (150W, 30°C, 2×5min; Ted Pella Biowave, Redding CA, USA). After washing with 0.01M PBS, secondary antibodies (AlexaFluor647 donkey anti-goat (1:600), Invitrogen, Waltham, MA, USA, AB_2762835; AlexaFluor488 donkey anti-rabbit (1:600), Invitrogen, AB_2762838) were added for 2h at room temperature, then washed and coverslipped with mounting medium with DAPI (Abcam).

For each animal, 3 serial sections were imaged through 5 counting frames within the dorsal hippocampus with a Zeiss LSM 700 (Baden-Wurttemberg, DE) or Leica TCS SPE laser scanning confocal microscope (Leica Microsystems, Inc, Deerfield, IL, USA) with a 20x objective lens. Z-stack images (20-25; 1μm step) were acquired and processed with FIJI (Schindelin et al., 2012; SCR_002285). Ionized calcium binding adaptor molecule 1-positive (Iba1+) microglia and glial fibrillary acidic protein-positive (GFAP+) astrocytes in the left dorsal hippocampus were counted blindly. Classification into activated and homeostatic astrocytes and microglia was performed using previously established criteria (Karperien et al., 2013; Wilhelmsson et al., 2006). The CORT-treated group was used as a reference to control for batch effects between staining and imaging sessions.

**Statistics:** qPCR sample size was determined to be N=4/group based on previous calculations (Locker et al., 2017). A larger N/group (see **Supplementary Table 1**) was used to account for DFP-related mortality (~25%) and statistical or technical outliers. Statistical outliers were identified using Grubb’s test (α=0.05) (GraphPad QuickCalcs: https://www.graphpad.com/quickcalcs/Grubbs1.cfm). LPS alone groups were excluded from analysis due to unexpectedly high cytokine responses compared to other LPS-treated groups within the study and historical data. mRNA data was analyzed in SigmaPlot v15 (Systat Software, Inc; SCR_003210). Based on previous studies and experimental hypotheses, we were interested in several specific, *a priori* comparisons: Control(s) v. CORT+LPS and CORT+DFP+LPS (to determine instigation of an inflammatory response), CORT+DFP v. CORT+DFP+LPS/CORT v. CORT+LPS (to determine an LPS-driven inflammatory response), and CORT+LPS v. CORT+DFP+LPS (to determine if the GW-relevant exposure to DFP contributes to a worsening inflammatory response). A 2x2 factorial two-way ANOVA (pretreatment [CORT or CORT+DFP] x exposure [saline or LPS]) on log-transformed values was performed initially to evaluate potential interactions between pretreatment x exposure, potential main effects for each factor, as well as for graphical representation of the data. This analysis found only liver *Osm* to have a significant interaction (*F =* 7.094, *P* = 0.017), but several significant main effects for pretreatment and/or exposure were identified (see **Supplementary Table 2)**. In order to determine the statistical significance for the *a priori* comparisons, the data were re-fit to a single factor one-way ANOVA analysis including the following groups: Saline (Control), CORT, CORT+LPS, CORT+DFP, and CORT+DFP+LPS, to perform post-hoc pairwise comparisons. Reported significance values in **Figure 1** are only the *a priori* comparison results based on the one-way ANOVA pairwise comparisons. MRI and cell counting data were analyzed using MATLAB (R2022a; The MathWorks, Inc, Natick, MA, USA; SCR_001622).

**Supplementary Table 1. Subject Characteristics**

| **Group** | **Count for qPCR** | **Count for MRI** | **Count for immunostaining** |
| --- | --- | --- | --- |
| Control (Saline injection only) | 5 | 4 | N/A |
| CORT only | 5 | 5 | 3 |
| LPS only | 5 | 5 | N/A |
| CORT+LPS | 5 | 5 | 3 |
| CORT+DFP | 7 | 7 | 3 |
| CORT+DFP+LPS | 7 | 4 | 3 |

**Supplementary Table 2. Two-way ANOVA main effect and interaction values for qPCR**

|  |  | ***Tnf*** | ***Il6*** | ***Ccl2*** | ***Il1b*** | ***Lif*** | ***Osm*** |
| --- | --- | --- | --- | --- | --- | --- | --- |
| **CORTEX** | Pretreatment | *F*=0.530*, P*=0.475 | *F*=3.303*, P*=0.086 | *F*=2.178*, P*=0.157 | *F*=0.142*, P*=0.711 | *F*=0.857*, P*=0.366 | *F*=0.422*, P*=0.524 |
|  | Exposure | *F*=7.418*, P*=0.013 | *F*=8.527*, P*=0.009 | *F*=4.282*, P*=0.053 | *F*=29.643*, P*<0.001 | *F*=0.312*, P*=0.583 | *F*=2.832*, P*=0.109 |
|  | Pretreatment x Exposure | *F*=0.361*, P*=0.555 | *F*=0.121*, P*=0.732 | *F*=1.451*, P*=0.244 | *F*=0.980*, P*=0.335 | *F*=1.647*, P*=0.215 | *F*=0.899*, P*=0.355 |
| **HIPPOCAMPUS** | Pretreatment | *F*=1.064*, P*=0.316 | *F*=20.671*, P*<0.001 | *F*=6.552*, P*=0.020 | *F*=1.243*, P*=0.279 | *F*=0.027*, P*=0.872 | *F*=1.827*, P*=0.192 |
|  | Exposure | *F*=10.858*, P*=0.004 | *F*=32.107*, P*<0.001 | *F*=6.552*, P*=0.020 | *F*=37.467*, P*<0.001 | *F*=0.731*, P*=0.403 | *F*=7.577*, P*=0.013 |
|  | Pretreatment x Exposure | *F*=0.084*, P*=0.775 | *F*=0.623*, P*=0.440 | *F*=1.649*, P*=0.215 | *F*=0.647*, P*=0.431 | *F*=0.003*, P*=0.954 | *F*=1.306*, P*=0.267 |
| **LIVER** | Pretreatment | *F*=3.387*, P*=0.082 | *F*=4.485*, P*=0.048 | *F*=0.761*, P*=0.394 | *F*=1.514*, P*=0.234 | *F*=6.146*, P*=0.023 | *F*=9.625*, P*=0.007 |
|  | Exposure | *F*=224.82*, P*<0.001 | *F*=319.68*, P*<0.001 | *F*=216.92*, P*<0.001 | *F*=529.04*, P*<0.001 | *F*=35.523*, P*<0.001 | *F*=111.81*, P*<0.001 |
|  | Pretreatment x Exposure | *F*=0.050*, P*=0.826 | *F*=0.278*, P*=0.605 | *F*=0.635*, P*=0.436 | *F*=1.466*, P*=0.242 | *F*=0.335*, P*=0.570 | *F*=7.094*, P*=0.017 |

**
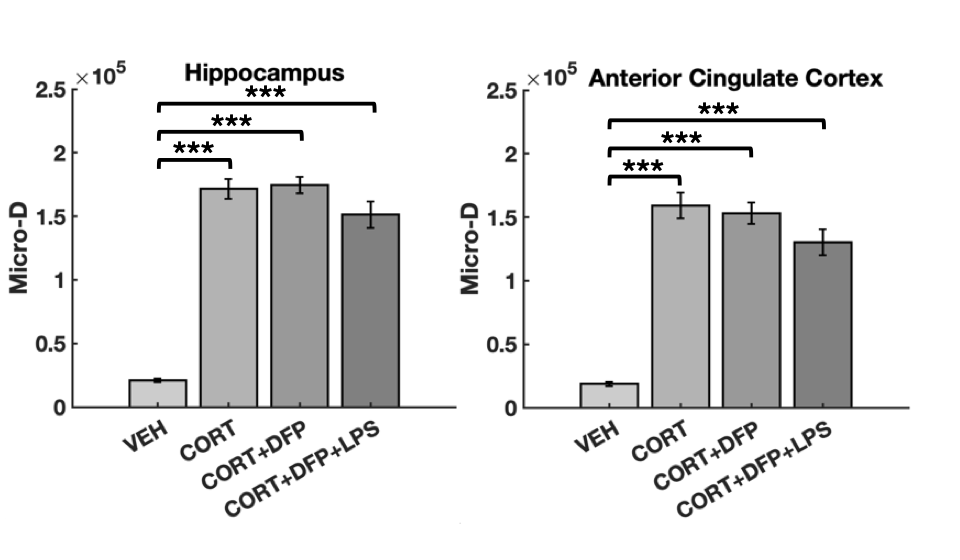
**

**Supplementary Figure 1.** Group-level comparison of microscale restricted diffusivity (micro-D) in each ROI comparing exposure groups to the vehicle-treated control group (VEH), *** p≤0.0005.

#### Supplementary References

Barrière, D. A., et al. "The SIGMA rat brain templates and atlases for multimodal MRI data

analysis and visualization." *Nature communications* 10.1(2019):5699.

Karperien, Audrey, et al. "Quantitating the subtleties of microglial morphology with fractal analysis." *Frontiers in cellular neuroscience* 7(2013):3.

Wilhelmsson, Ulrika, et al. "Redefining the concept of reactive astrocytes as cells that remain

within their unique domains upon reaction to injury." *Proceedings of the National Academy of Sciences* 103.46(2006):17513-17518.

Schindelin Johannes, et al. “Fiji: an open-source platform for biological-image analysis.” *Nature Methods* 9(2012):676-82.
